# Gait kinematics and kinetics in patients with different grades of meniscus injury: A cross-sectional study

**DOI:** 10.1371/journal.pone.0347740

**Published:** 2026-04-27

**Authors:** Xinya Lan, Yaping Li, Jiaxin Gao, Yongyi Xu, Qingping Su, Pan Xu, Jianhao Chen, Zhonghua Lin, Cai Jiang

**Affiliations:** 1 Rehabilitation Medicine Center, Fuzhou University Affiliated Provincial Hospital, Fuzhou, China; 2 Shengli Clinical Medical College of Fujian Medical University, Fuzhou, China; 3 The Third Affiliated Hospital of Guangzhou University of Chinese Medicine, Guangzhou, China; University of Tehran, IRAN, ISLAMIC REPUBLIC OF

## Abstract

Meniscal injuries are common and can alter knee biomechanics, increasing the risk of osteoarthritis. This study investigated the effects of unilateral meniscal injuries of different Stoller grades on gait kinematics and kinetics. A total of 158 participants were stratified by MRI into three groups: control group(Grade 0, n = 51), Grade I–II (n = 54), and Grade III (n = 53). Three-dimensional motion capture synchronized with force platforms was used to assess peak sagittal-plane joint angles, joint moments, and ground reaction forces. Multivariate analysis of covariance was applied to adjust for body mass index, Lysholm score, and walking speed. Compared with healthy controls, injured participants demonstrated reduced knee flexion, hip extension, and lower extremity joint moments, along with increased ankle dorsiflexion, knee extension, and hip flexion angles; anterior, posterior, and lateral ground reaction forces were also significantly decreased. Although no significant differences in joint moments or ground reaction forces were observed between Grade I–II and Grade III groups, deviations in joint angles increased gradually with the severity of injury. The most pronounced changes were seen in the knee extension angle, which rose by 309.0% in Grade III compared with the control group, and the hip extension angle, which decreased by 53.3% in Grade III compared with the control group. A 16.5% reduction in the knee flexion angle was also observed. These findings indicate that even mild meniscal injuries produce substantial gait kinetic deficits, while kinematic alterations become more pronounced with higher-grade injuries. The study highlights the value of integrating Stoller grading with objective gait analysis to identify functional impairments not captured by patient-reported outcomes. This comprehensive approach provides a biomechanical basis for early assessment and individualized rehabilitation strategies, supporting knee function preservation and potentially slowing long-term degenerative changes.

## Introduction

The knee meniscus plays a crucial role in joint stability, load distribution, and impact absorption, serving as an essential structure for maintaining knee function during dynamic activities such as walking [1]. Meniscus injuries are highly prevalent in the general population. Magnetic resonance imaging (MRI) studies indicate that the incidence of meniscus injury can reach 35% in asymptomatic middle-aged and elderly individuals, and up to 67% in those over 65 years of age [2,3]. Such injuries, particularly complex tears or meniscal extrusion, substantially increase the risk of knee osteoarthritis (OA) [2,4]. The biomechanical consequences mainly include increased joint contact pressure and altered joint kinematics, such as posterior tibial translation and excessive internal rotation, which disrupt normal load distribution and promote cartilage degeneration [5,6]. Additionally, inflammatory responses can further amplify these effects, especially in obese or elderly patients, accelerating OA progression [7].

In recent years, three-dimensional gait analysis has been widely applied in evaluating knee function [8–10]. It enables quantification of lower limb motor function and compensatory mechanics, providing objective indicators for functional assessment of meniscus injuries. However, existing studies have primarily focused on gait changes before and after surgical interventions, such as meniscectomy, with less attention given to cross-sectional comparisons under natural injury conditions [6]. Most cross-sectional studies have disproportionately included cases with concomitant anterior cruciate ligament (ACL) injuries, which may substantially confound gait outcomes, as ACL deficiency is known to alter anterior–posterior stability, neuromuscular control strategies, and joint loading patterns during walking [11–13]. The presence of ACL tears can therefore produce compensatory kinematic and kinetic adaptations that differ fundamentally from those caused by isolated meniscal pathology, making it difficult to distinguish the independent biomechanical contribution of the meniscus lesion itself. Consequently, studies specifically focusing on isolated meniscus injuries are essential to accurately characterize the functional impact attributable solely to meniscal damage. The Stoller grading system, based on MRI characteristics, is widely used clinically to assess the severity of meniscus injuries [14]. Clinically, Stoller Grade I–II lesions are generally considered to represent intrameniscal degenerative signal changes without definite surface disruption and are often associated with early or chronic degenerative processes, whereas Grade III lesions indicate a definite meniscal tear extending to the articular surface and are more frequently accompanied by mechanical symptoms such as locking or catching. Although the Stoller system provides a reliable pathological basis, studies combining it with gait analysis to evaluate functional outcomes are still scarce.

This study aims to address this gap by investigating the biomechanical effects of Stoller-graded meniscus injuries on gait parameters of the affected limb during walking. We hypothesize that, compared with patients with Grade I–II injuries, those with Grade III meniscus injuries will exhibit more pronounced impairments in gait kinematics and kinetics, with both groups differing significantly from healthy controls. By integrating Stoller grading with gait analysis, this study seeks to elucidate the functional consequences of varying meniscus injury severity, offering insights for the development of targeted rehabilitation strategies and the mitigation of long-term joint degeneration.

## Methods

### Study design

This single-center, prospective cross-sectional study was conducted at Fuzhou University Affiliated Provincial Hospital between June 2023 and December 2024. The study was carried out in accordance with the Declaration of Helsinki and was approved by the Medical Ethics Committee of Fuzhou University Affiliated Provincial Hospital (No. K2023-05–073; approved on May 26, 2023). Written informed consent was obtained from all participants prior to enrolment.

### Sample size calculation

Sample size estimation was performed using G*Power 3.1 software [15]. Based on a MANCOVA design with three groups, 26 dependent variables, and three covariates, and referring to medium-to-large effect sizes reported in the biomechanics literature of knee disorders [16–18], a conservative Cohen’s f² = 0.25 was chosen. With an α of 0.05, statistical power (1-β) of 0.80, and a 95% confidence interval, the minimum required sample size was calculated to be 81 participants. To account for potential dropouts and ensure adequate statistical power, a total of 158 participants were ultimately included. We acknowledge that this choice may result in slightly optimistic statistical power for detecting differences specifically between Grade I–II and Grade III groups. However, given the cross-sectional design of our study and the overall sample size, this approach remains reasonable to detect clinically meaningful differences across the three groups.

### Participants

All participants were screened according to strict inclusion and exclusion criteria and categorized based on MRI Stoller gradingcombined with clinical symptoms into control group(Grade 0, n = 51), Grade I–II (n = 54), and Grade III (n = 53) [14]. It should be noted that, in clinical MRI reports and research studies, Grade I and II meniscus injuries are often collectively referred to as “Grade I–II lesions” because both present as signal changes confined within the meniscus without involving the articular surface, and they largely overlap in clinical presentation and conservative management strategies [19–21]. In routine radiological reporting at our institution, findings were documented as Grade I, Grade I–II, or Grade II, without standardized subclassification or consistent quantitative differentiation between isolated Grade I and isolated Grade II lesions. Consequently, the exact number of purely Grade I versus purely Grade II cases was not systematically recorded in the database at the time of data collection. Inclusion criteria for meniscus injury patients: clinically confirmed unilateral meniscus injury confirmed by both MRI and clinical examination; clear consciousness; adequate cognitive, comprehension, and communication abilities to comply with experimental procedures. Exclusion criteria: pregnancy; concomitant ACL or other ligament injuries; other musculoskeletal disorders (e.g., ankle sprain, femoroacetabular impingement, or non-specific low back pain); limb instability caused by non-traumatic factors, assessed using Lachman test and Anterior Drawer test; neurological or vestibular disorders affecting balance (including stroke, Parkinson’s disease, vestibular neuritis, etc.); prior treatment for meniscus injury; withdrawal from the study before completion. The control group consisted of healthy volunteers recruited from the surrounding community. Inclusion criteria for the control group: no history of knee trauma or surgery; MRI confirmation of no meniscus injury or other knee pathology in the dominant limb; participants reported no current knee pain or symptoms. It should be pointed out that, while gait parameters were analyzed from the injured limb for participants in the injury groups, the dominant limb was selected for analysis in the control group.

### Instrumentation

Data were collected using the SMART-D 400 infrared motion capture system (BTS, Italy). The system included eight infrared cameras (operating at 200 Hz), two synchronous cameras (BTS eVixta, 50 Hz), four force platforms (BTS P6000D, 1000 Hz), a data converter, and a computer system. Three-dimensional motion data were obtained by capturing the trajectories of reflective markers placed on participants’ bodies using infrared high-speed cameras, allowing comprehensive gait parameter analysis.

### Testing procedure

Prior to testing, reflective objects were removed, doors and windows were closed, curtains drawn, and room temperature adjusted. The system was calibrated to ensure all cameras and the computer system were properly connected and positioned. Participants removed shoes and socks and wore appropriate clothing. Anthropometric measurements including height, weight, leg length, pelvic height, pelvic width, knee width, and ankle width were recorded. Following the Davis protocol based on the Helen Hayes marker set [22], 22 markers were placed on each participant: C7, bilateral acromion, bilateral anterior superior iliac spine, bilateral greater trochanter, bilateral lateral femoral condyle, bilateral fibular head, bilateral lateral malleolus, bilateral calcaneus, bilateral fifth metatarsal, and sacral midpoint. Additional small rods with markers were fixed at the midpoints between the greater trochanter and lateral femoral condyle, and between the fibular head and lateral malleolus. For static model establishment, participants stood naturally with arms at their sides and feet shoulder-width apart. Participants then walked back and forth along a 10-meter path at a self-selected speed, with laser timing gates positioned 5 meters apart on both sides of the force platforms to determine walking speed. Participants practiced several trials to familiarize themselves with the equipment and establish their self-selected walking speed. Three valid trials were collected for each limb. Trials were considered valid if the target limb fully contacted one force platform among four and walking speed remained within ±5% of the self-selected speed. Contact was deemed acceptable when the contralateral limb contacted a different force plate, provided that both limbs did not simultaneously contact the same force platform.

### Data processing

Three-dimensional marker coordinates were reconstructed using the BTS SMART Tracker system and synchronized with ground reaction forces (GRF) data. Raw coordinate data were filtered using a fourth-order zero-lag Butterworth low-pass filter with a cutoff frequency of 6 Hz (Visual 3D, version 5, C-Motion, Inc., Germantown, MD). Kinematic parameters of the ankle, knee, and hip were calculated using a joint coordinate system approach, considering six degrees of freedom for each segment [23]. GRF was normalized to body mass (N·kg ⁻ ¹). Anthropometric, kinematic, and GRF data were used to calculate joint moments of the ankle, knee, and hip in the sagittal plane using standard inverse dynamics equations. Joint moments were normalized to body weight and expressed in Nm·kg ⁻ ¹. Parameters of interest included sagittal plane peak joint angles (hip and knee flexion/extension, ankle dorsiflexion/plantarflexion), sagittal plane peak joint moments (hip and knee flexion/extension moments, ankle dorsiflexion/plantarflexion moments), and GRF parameters (vertical GRF first peak, second peak, and trough, as well as maximum anterior, posterior, medial, and lateral GRF).

### Statistical analysis

Data were analyzed using IBM SPSS Statistics 26.0. Continuous variables conforming to normal distribution were expressed as mean ± standard deviation ($\bar{x}$ ± s); non-normally distributed variables were presented as median (interquartile range) M (P25, P75). Baseline characteristics and normally distributed gait speed data were analyzed using one-way ANOVA, while non-normally distributed data were analyzed using the Kruskal–Wallis test. Categorical data were analyzed using the chi-square test. Statistical significance was defined as *P* < 0.05. Multivariate analysis of covariance (MANCOVA) was employed to detect differences in gait parameters between groups, with Lysholm score, body mass index (BMI), and gait speed included as covariates. Sensitivity analyses were performed by re-running MANCOVA models with alternative covariate specifications (excluding gait speed and excluding Lysholm score). Furthermore, Collinearity diagnostics were conducted, and variance inflation factors were calculated to assess multicollinearity among covariates. Bonferroni post-hoc tests were applied for multiple comparisons among the three groups, with *P* < 0.05 considered statistically significant.

## Results

### Demographic characteristics

Significant differences were observed among the three groups in BMI, Lysholm score, and gait speed (*P* < 0.05), whereas no significant differences were found in sex, weight, height, age, or exercise habits (*P* > 0.05) (Table 1). Multivariate testing (Table 2) showed that the intercept effect was significant in the model (F = 13.633, *P* < 0.001). BMI (F = 2.038, *P* = 0.010), gait speed (F = 3.250, *P* < 0.001), and injury severity (F = 7.077, *P* < 0.001) all had statistically significant effects on the dependent variables, while Lysholm score did not show a significant effect (F = 1.157, *P* = 0.304).

**Table 1:** Baseline characteristics of study participants(n = 158).

Variables	control group (n = 51)	Grade I–II injury group (n = 54)	Grade III injury group (n = 53)	F	*P*
Gender (n %)				2.051^a^	0.152
Male	29 (18.4)	30 (19.0)	29 (18.4)		
Female	22 (14.0)	24 (15.1)	24 (15.1)		
Affected side (%)				–	–
Left	–	23 (21.5)	24 (22.4)		
Right	–	31 (29.0)	29 (27.1)		
Age (years)	50.20 ± 23.15	46.37 ± 13.44	53.23 ± 13.38	2.143^b^	0.121
Height (cm)	167.12 ± 10.93	166.72 ± 8.40	163.77 ± 8.58	2.010^b^	0.137
Weight (kg)	63.63 ± 7.99	67.75 ± 7.58	66.16 ± 11.58	2.644^b^	0.074
BMI (kg/m²)	22.79 ± 2.61	24.29 ± 1.84	24.58 ± 3.38	6.620^b^	**0.002**
Exercise habits (n %)				2.051^a^	0.152
No	32 (20.3)	25(15.8)	31 (21.0)		
Yes	19 (12.0)	29 (18.4)	22 (13.2)		
Disease duration	–	11.00 ± 18.35	9.05 ± 17.20	–	–
Lysholm score	100.00 (100.00,100.00)	50.00 (40.00,50.00)	44.00 (39.00,50.00)	107.313^c^	**<0.001**
VAS score	–	4.00 (2.00,6.00)	5.00 (3.00,6.00)	–	–
Gait speed(m/s)	1.13 ± 0.18	0.91 ± 0.11	0.89 ± 0.18	19.401^b^	**<0.001**

BMI body mass index.

^a^Chi-Square Test, Values in bold are statistically significant, i.e., *p*-values < 0.05.

^b^one-way ANOVA, Values in bold are statistically significant, i.e., *p*-values < 0.05.

^c^Kruskal-Walls Test, Values in bold are statistically significant, i.e., *p*-values < 0.05.

**Table 2:** MANOVA Test Results (Wilks’ Lambda).

Effect	Statistic Value	F	Hypothesis df	Error df	*P*
Intercept	0.341	13.633	19	134	**<0.001**
BMI	0.776	2.038	19	134	**0.010**
Lysholm score	0.859	1.157	19	134	0.304
Gait speed	0.685	3.250	19	134	**<0.001**
Degree of Injury	0.249	7.077	38	268	**<0.001**

BMI body mass index.

Values in bold are statistically significant, i.e., *p*-values < 0.05.

### Peak joint angles

MANCOVA results (Table 3) demonstrated significant intergroup differences in peak joint angles. Peak ankle plantarflexion angle(F = 3.406, *P* = 0.006), peak ankle dorsiflexion angle(F = 28.300, *P* < 0.001), peak knee flexion angle(F = 18.191, *P* < 0.001), peak knee extension angle(F = 135.938, *P* < 0.001), peak hip flexion angle(F = 12.477, *P* < 0.001), and peak hip extension angle(F = 56.862, *P* < 0.001) all showed highly significant differences between groups. Bonferroni post-hoc analysis indicated that, compared with the control group, patients with meniscus injuries exhibited significant kinematic alterations. Specifically, Peak ankle plantarflexion angle was reduced by 9.0% in the Grade III group compared with the control group (d = 0.55, *P* = 0.007) and by 9.8% compared with the Grade I–II group (d = 0.73, *P* = 0.002). Peak ankle dorsiflexion angle increased significantly, with a 35.6% increase in the Grade I–II group (d = −1.97, *P* < 0.001) and a 45.9% increase in the Grade III group (d = −0.52, *P* = 0.036) relative to the control group. In the knee joint, peak knee flexion angle decreased significantly by 12.2% in the Grade I–II group (d = 1.33, *P* < 0.001) and by 16.5% in the Grade III group (d = 1.73, *P* < 0.001) compared with the control group. While peak knee extension angle increased significantly by 170.8% in the Grade I–II group (d = −3.75, *P* < 0.001) and by 309.0% in the Grade III group (d = −4.72, *P* < 0.001) compared with the control group, and by 80.9% in the Grade III group compared with the Grade I–II group (d = −1.98, *P* < 0.001). For the hip joint, peak hip flexion angle increased by 16.1% in the Grade I–II group (d = −1.29, *P* < 0.001), by 25.2% in the Grade III group (d = −1.52, *P* < 0.001) compared with the control group, and by 7.9% in the Grade III group compared with the Grade I–II group (d = −0.47, *P* = 0.013). Peak hip extension angle decreased significantly by 30.2% in the Grade I–II group (d = 1.72, *P* < 0.001), by 53.3% in the Grade III group (d = 3.38, *P* < 0.001) relative to the control group, and by 33.0% in the Grade III group compared with the Grade I–II group (d = 1.55, *P* < 0.001).

**Table 3:** Comparison of gait parameters among the three groups (MANCOVA).

Gait parameter	Control group(mean±SD	Grade I-II (mean±SD)	Grade III (mean±SD)	F (*p*-value)	Absolute difference		
	(n = 51)	(n = 54)	(n = 53)				
					Control vs. Grade I-II	Control vs. Grade III	Grade I-II vs. Grade III
					d,(*p*-value)	d,(*p*-value)	d,(*p*-value)
**Joint angles (°)**							
Peak ankle plantarflexion angle	21.93 ± 3.46	22.09 ± 2.20	20.12 ± 3.14	**3.406(0.006)**	−0.06 (1.000)	**0.55 (0.007)**	**0.73 (0.002)**
Peak ankle dorsiflexion angle	12.74 ± 2.94	17.28 ± 1.93	18.59 ± 2.99	**28.300(<0.001)**	**−1.84(<0.001)**	**−1.97 (<0.001)**	**−0.52 (0.036)**
Peak knee flexion angle	68.70 ± 4.96	60.32 ± 7.34	57.35 ± 7.81	**18.191(<0.001)**	**1.33 (<0.001)**	**1.73(<0.001)**	0.39(0.078)
Peak knee extension angle	1.44 ± 0.54	3.90 ± 0.75	5.89 ± 1.21	**135.938(<0.001)**	**−3.75(<0.001)**	**−4.72 (<0.001)**	**−1.98(<0.001)**
Peak hip flexion angle	34.09 ± 2.44	39.58 ± 5.43	42.69 ± 7.54	**12.477(<0.001)**	**−1.29(<0.001)**	**−1.52 (<0.001)**	**−0.47 (0.013)**
Peak hip extension angle	7.77 ± 1.43	5.42 ± 1.30	3.63 ± 0.99	**56.862(<0.001)**	**1.72(<0.001)**	**3.38 (<0.001)**	**1.55(<0.001)**
**Joint moments (N·m/kg)**							
Peak ankle plantarflexion moment	1.50 ± 0.23	1.54 ± 0.33	1.41 ± 0.26	1.831 (0.110)	−0.14 (1.000)	0.37 (0.318)	0.44 (0.061)
Peak ankle dorsiflexion moment	0.26 ± 0.14	0.19 ± 0.09	0.18 ± 0.09	**4.331 (0.001)**	**0.60 (0.001)**	**0.68 (<0.001)**	0.11 (1.000)
Peak knee flexion moment	0.52 ± 0.23	0.40 ± 0.27	0.46 ± 0.29	1.204 (0.310)	0.48 (0.056)	0.23 (0.650)	−0.21 (0.766)
Peak knee extension moment	0.34 ± 0.27	0.25 ± 0.20	0.21 ± 0.16	**3.286 (0.008)**	0.38 (0.078)	**0.59 (0.007)**	0.22 (1.000)
Peak hip flexion moment	0.56 ± 0.30	0.43 ± 0.29	0.38 ± 0.22	**8.183 (<0.001)**	**0.44 (0.044)**	**0.69 (0.002)**	0.19 (0.956)
Peak hip extension moment	0.85 ± 0.26	0.64 ± 0.20	0.62 ± 0.16	**11.402 (<0.001)**	**0.91 (<0.001)**	**1.07 (<0.001)**	0.11 (1.000)
**Ground reaction forces (% body weight)**							
Vertical GRF first peak	94.73 ± 12.52	90.62 ± 10.64	92.29 ± 9.48	0.996 (0.422)	0.35 (0.167)	0.22(0.770)	−0.17 (1.000)
Vertical GRF second peak	104.37 ± 9.11	102.23 ± 8.58	101.37 ± 9.46	1.226(0.300)	0.24 (0.682)	0.32(0.277)	0.10 (1.000)
Vertical GRF valley	74.23 ± 9.39	75.60 ± 9.49	76.34 ± 8.90	1.127 (0.349)	−0.15 (1.000)	−0.23 (0.742)	−0.08 (1.000)
Peak anterior GRF	15.31 ± 3.45	12.57 ± 2.83	12.45 ± 3.73	**11.025(<0.001)**	**0.87 (<0.001)**	**0.80 (<0.001)**	0.04 (1.000)
Peak medial GRF	4.80 ± 1.80	5.26 ± 1.16	5.32 ± 1.20	2.200 (0.057)	−0.31 (0.306)	−0.34 (0.197)	−0.05 (1.000)
Peak posterior GRF	12.86 ± 3.64	10.41 ± 2.77	10.40 ± 3.10	**7.988 (<0.001)**	**0.76 (<0.001)**	**0.73 (<0.001)**	0.01 (1.000)
Peak lateral GRF	2.97 ± 1.47	2.05 ± 1.00	2.16 ± 0.85	**5.399 (<0.001)**	**0.74 (<0.001)**	**0.68 (0.001)**	−0.12 (1.000)

GRF ground reaction forces.

^a^MANCOVA with adjustment for lysholm score, gait speed, BMI,Values in bold are statistically significant, i.e., *p*-values < 0.05 for MANCOVAs.

^b^Bonferroni correction was used in post-hoc comparisons across the three groups. Values in bold are statistically significant, i.e., *p*-values < 0.05 for post-hoc tests.

### Peak joint moments

MANCOVA results (Table 3) revealed significant intergroup differences in peak joint moments. Specifically, peak ankle dorsiflexion moment (F = 4.331, *P* = 0.001), peak knee extension moment (F = 3.286, *P* = 0.008), peak hip flexion moment (F = 8.183, *P* < 0.001), and peak hip extension moment (F = 11.402, *P* < 0.001) all differed significantly between groups, whereas peak ankle plantarflexion moment (F = 1.831, *P* = 0.110) and peak knee flexion moment (F = 1.204, *P* = 0.310) did not show significant intergroup differences (*P* > 0.05). Bonferroni post-hoc analysis indicated that, compared with the control group, patients with meniscus injuries exhibited significant reductions in several parameters. Peak ankle dorsiflexion moment decreased by 26.9% in the Grade I–II group (d = 0.60*, P* = 0.001) and by 30.8% in the Grade III group (d = 0.68, *P* < 0.001) relative to the control group. Peak knee extension moment was reduced by 38.2% in the Grade III group compared with the control group (d = 0.59, *P* = 0.007). Peak hip flexion moment decreased by 23.2% in the Grade I–II group (d = 0.44, *P* = 0.044) and by 32.1% in the Grade III group (d = 0.65, *P* = 0.002) compared with the control group. Peak hip extension moment decreased by 24.7% in the Grade I–II group (d = 0.91, *P* < 0.001) and by 27.1% in the Grade III group (d = 1.07, *P* << 0.001). Notably, all significant differences were observed between the control group and the injury groups (both Grade I–II and Grade III), while no significant differences were detected between the Grade I–II and Grade III groups for any joint moment parameter (*P* > 0.05).

### GRF

Regarding GRF, MANCOVA results (Table 3) showed significant intergroup differences in anterior GRF (F = 11.025, *P* < 0.001), posterior GRF (F = 7.988, *P* < 0.001), and lateral GRF (F = 5.399, *P* < 0.001), whereas no significant differences were observed in vertical GRF parameters, including first peak, second peak, and valley(*P* > 0.05). Bonferroni post-hoc analysis indicated that, compared with the control group, meniscus injury groups exhibited significant reductions in these parameters. Anterior GRF decreased by 17.9% in the Grade I–II group (d = 0.87, *P* < 0.001) and by 18.7% in the Grade III group (d = 0.80, *P* < 0.001) relative to the control group. Posterior GRF decreased by 19.1% in the Grade I–II group (d = 0.76, *P* < 0.001) and by 19.1% in the Grade III group (d = 0.73, *P* < 0.001). Lateral GRF decreased by 31.0% in the Grade I–II group (d = 0.74, *P* < 0.001) and by 27.3% in the Grade III group (d = 0.68, *P* = 0.001) compared with the control group. Importantly, all significant differences were found between the control group and the injury groups, whereas no significant differences were observed between the Grade I–II and Grade III groups for any GRF parameter (*P* > 0.05).

### Sensitivity analysis and multicollinearity assessment

Sensitivity analyses were performed by sequentially removing gait speed and Lysholm score from the covariate structure. The overall multivariate effect of injury severity remained statistically significant across all models (all *p* < 0.001) (Table 4).

**Table 4:** Multivariate Sensitivity Analyses of the MANCOVA Models.

Model	Wilks’Λ	F (df1, df2)	*p*-value
Full model	0.249	7.08 (38, 268)	<0.001
Without gait speed	0.248	7.15 (38, 270)	<0.001
Without Lysholm score	0.094	16.08 (38, 270)	<0.001

a Full model adjusted for BMI, Lysholm score, and gait speed.

b Wilks’Λ was used to assess the overall multivariate group effect of injury severity.

At the univariate level, the majority of biomechanical parameters retained their statistical significance. Medial shear force demonstrated modest sensitivity to covariate adjustment, reaching statistical significance after removal of individual covariates; however, this did not alter the overall interpretation of the results. Collinearity diagnostics demonstrated that all variance inflation factors were below 5.0, and tolerance values exceeded 0.20 (range: 1.14–4.20) (Table 5).

**Table 5:** Multicollinearity Diagnostics for Covariates in the MANCOVA Model.

Variable	Tolerance	VIF
BMI	0.878	1.139
Gait speed	0.655	1.527
Lysholm score	0.238	4.198

VIF, variance inflation factor.

## Discussion

Our findings demonstrate that meniscus injury significantly alters the gait biomechanics of the affected limb. Both Grade I–II and Grade III patients exhibited marked differences compared with healthy controls, partially confirming our hypothesis. However, the hypothesis that Grade III injuries would result in more severe kinetic dysfunction was not supported; only joint kinematics could distinguish between the two injury severities. By revealing complex nonlinear interactions between Stoller grading and gait biomechanics, this study clarifies the impact of meniscal lesions on kinematic and kinetic gait parameters.

Multivariate analysis indicated that injury severity had a statistically significant effect on gait parameters, suggesting that Stoller grading reflects not only imaging abnormalities but also functional deficits. Li et al. found through three-dimensional gait analysis that Stoller grade correlates significantly with knee kinematic parameters, indicating that imaging severity reflects both anatomical and functional alterations, such as reduced knee flexion-extension range and increased internal rotation [24]. In contrast, our study found no significant association between Lysholm functional scores and gait parameters, highlighting that subjective functional reports may mask objective biomechanical deficits. Tornbjerg et al. observed in patients with meniscus tears that structural lesions, such as tear type or cartilage damage, were not significantly associated with preoperative self-reported pain or function, suggesting that subjective scores may underestimate the actual functional risk of early injury [25]. Further, MacFarlane et al. reported a weak association between patient-reported knee swelling and MRI-defined effusion-synovitis, supporting the dissociation between symptoms and structural abnormalities [26]. Therefore, clinical assessment should prioritize objective gait analysis tools rather than relying solely on patient-reported measures.

In our multivariate model, gait speed and BMI significantly influenced gait kinetics. Similar observations were reported by Wang et al. and Nilsson, who noted that faster walking speeds substantially increase vertical ground reaction forces and knee joint contact forces, amplifying gait asymmetry [27,28]. Harding et al. further indicated that higher BMI is closely associated with knee loading, potentially altering joint moment patterns and muscle recruitment strategies, thereby enhancing compensatory motor control and increasing the risk of osteoarthritis [29,30]. Gait speed may theoretically lie on the causal pathway between meniscal injury and biomechanical alterations. Adjusting for such a mediator could potentially obscure true effects. Therefore, we performed sensitivity analyses with and without gait speed adjustment. The persistence of significant group differences across models suggests that observed biomechanical alterations were not solely driven by walking speed differences. Adjusting for these covariates in the current study helps isolate compensatory mechanisms related to the injury itself from general effects of speed or body mass, enhancing the robustness and clinical interpretability of our conclusions.

According to inverse dynamics theory, joint moments depend on the interaction of ground reaction forces, moment arms determined by joint angles, and segmental inertial properties [31]. The absence of significant differences in gait kinetics between the Grade I–II and Grade III groups suggests that patients with varying injury severities may systematically adjust joint angles to modify moment arms, thereby mitigating joint moments. Specifically, during stance, the ankle exhibits increased dorsiflexion and reduced plantarflexion, shortening the posterior ground reaction force moment arm and reducing plantarflexion moments and propulsive power. Restricted knee flexion limits anterior tibial translation, decreasing the lever arm for knee extension moments, a “stiff-knee” pattern also observed in ACL-deficient gait stabilization strategies [32,33]. At the hip, excessive flexion and limited extension alter lower limb orientation, reducing reliance on extensor recruitment during terminal stance, consistent with compensatory patterns reported in early knee osteoarthritis [34]. These systematic angle adjustments shorten GRF moment arms, reducing sagittal plane joint moments, similar to the kinematic compensations observed in posterior root meniscus tear models [5].

Beyond structural adaptations, psychosocial factors warrant attention. Tang et al. reported that kinesiophobia in meniscus-injured patients correlates positively with injury severity, pain intensity, and balance deficits, and negatively with self-efficacy [35]. This suggests that patients with Grade I–II injuries may stiffen the knee and limit ankle push-off due to pain and fear of movement, exhibiting kinetic patterns similar to Grade III patients. Henriksen et al. further supported this compensatory mechanism, showing experimentally that pain directly induces reductions in knee adduction and flexion-extension moments in healthy subjects [36]. Although Grade III patients exhibited slightly higher VAS scores than Grade I–II patients, this did not result in further reductions in joint moments. Pain inhibition likely remains the primary factor limiting weight-bearing in both groups, and the larger tear in Grade III may not have caused additional functional limitation. Thus, pain and instability from meniscus injury prompt protective gait strategies that reduce GRF and moment arm lengths, decreasing ankle, knee, and hip moments. However, these strategies come at the cost of walking efficiency and may accelerate degenerative changes due to increased loading rates.

Compared with healthy controls, patients with Grade I–II and Grade III meniscus injuries exhibited significantly reduced peak knee flexion angles and increased peak knee extension angles, reflecting an adaptive but maladaptive “stiff-knee” posture consistent with a compensatory stiffening strategy aimed at enhancing joint stability. This pattern aligns with the “quadriceps avoidance” theory, whereby patients reduce knee flexion to minimize anterior tibial translation and protect the meniscus [37,38]. This finding aligns with the systematic review by Grassi et al.[39], which emphasized that the medial meniscus, particularly the posterior horn, serves as a key secondary stabilizer resisting anterior tibial translation, whereas the lateral meniscus primarily constrains dynamic rotation and axial translation. At the hip, patients—especially those with Grade III injuries—demonstrated increased flexion angles but restricted extension, consistent with Ferber et al.[40], who observed augmented hip flexion in patients with chronic anterior cruciate ligament deficiency. This redistribution of sagittal plane motion toward proximal musculoskeletal structures likely represents a compensatory strategy to maintain gait propulsion in the context of knee dysfunction. At the ankle, Grade III patients exhibited increased dorsiflexion and reduced plantarflexion, highlighting a further redistribution of sagittal plane motion. As Winter described, reduced plantarflexion shortens the lever arm, thereby diminishing propulsive force [31]. Collectively, these patterns suggest that as meniscal degeneration progresses, patients adopt a stiffer gait with reduced propulsion. Early identification of these joint angle alterations may therefore serve both as a biomarker of disease progression and as a targeted focus for gait retraining interventions.

The findings of this study indicate that gait impairments resulting from meniscal lesions cannot be attributed to a single joint or isolated variable, highlighting important clinical implications for the design of rehabilitation strategies for patients with meniscus injuries. Notably, even patients with mild Stoller Grade I–II injuries exhibited GRF and joint moment deficits similar to those seen in Grade III patients, suggesting that functional impairments may occur earlier than previously recognized, even in cases classified as “mild” by MRI. Furthermore, the progressive deviations in joint angles observed with increasing injury severity underscore the need for targeted interventions, particularly aimed at restoring hip extension, knee flexion, and ankle plantarflexion to prevent further deterioration in severe cases. Consequently, patients with MRI-confirmed “mild” abnormalities should still undergo functional assessments, as subclinical stiffness may indicate preclinical functional deficits. For example, Rennie et al. reported that 48% of asymptomatic athletes’ knees exhibited meniscal extrusion, which was significantly associated with meniscal tears [41].

Beyond laboratory-based 3D motion analysis, cost-effective assessment tools such as the timed up-and-go test or single-leg squat test can be used to screen for gait abnormalities in resource-limited settings [42,43]. Rehabilitation programs should incorporate closed-chain exercises, such as controlled squats, to address deficits in knee flexion and reduced propulsion, consistent with Frizziero et al. [44], who advocated early range-of-motion training following meniscectomy. Potential strategies targeting insufficient quadriceps neural activation, such as high-load resistance training and neuromuscular electrical stimulation, have been shown in previous studies to effectively enhance neural drive and improve muscle strength [45].

During follow-up, real-time monitoring of ground reaction forces and joint angles using pressure insoles or wearable sensors, together with periodic imaging and monitoring of inflammatory markers such as IL-6, as well as screening for kinesiophobia, can support personalized care by allowing dynamic evaluation of progress and adjustment of interventions [42,46]. By leveraging biomechanical markers, rehabilitation can facilitate early normalization of load distribution, potentially slowing the progression of osteoarthritis.

This study is among the few that stratify patients with isolated meniscus injuries based on Stoller grading. By employing multivariate models to adjust for covariates such as gait speed and BMI, the study enhances both the robustness and clinical interpretability of its findings. However, several limitations should be acknowledged. To achieve adequate statistical interpretability, Grade I and II injuries were combined into a single group. While this approach reflects common clinical reporting practice and the substantial overlap in structural characteristics and conservative management strategies, it may obscure subtle biomechanical differences between early degenerative stages. Importantly, because MRI findings were documented as Grade I, Grade I–II, or Grade II without consistent standardized subclassification, the precise distribution of isolated Grade I versus isolated Grade II lesions was not systematically recorded. As a result, separate subgroup analyses between Grade I and Grade II were not feasible within the present dataset. In addition, several structural and anatomical factors were not incorporated into the present analysis. The distribution of medial versus lateral meniscal injuries was not systematically recorded in the study database; therefore, all unilateral meniscal injuries were pooled for analysis. Given the distinct biomechanical roles of the medial and lateral menisci, this approach may limit compartment-specific interpretation. Furthermore, detailed tear morphology, meniscal extrusion status, cartilage condition, and lower limb alignment parameters were not included in the predefined data collection framework. As the study was designed to examine functional gait alterations across Stoller grades in isolated meniscal injury, these structural variables were outside the primary scope of investigation. Their omission should be considered when interpreting the findings. Also, the cross-sectional design does not allow causal inference. Specifically, the present design cannot distinguish whether the observed gait alterations are a direct consequence of meniscal injury or represent pre-existing biomechanical patterns that may have contributed to the development or progression of meniscal degeneration. One plausible scenario is that pain, instability, and kinesiophobia following meniscal injury induce protective guarding strategies, resulting in the stiffened gait pattern observed in the current cohort. Conversely, pre-existing biomechanical abnormalities such as altered sagittal plane loading patterns, quadriceps weakness, or subtle malalignment may chronically increase meniscal stress and predispose individuals to degenerative lesions, particularly in Stoller Grade I–II cases. A bidirectional model is also likely, in which predisposing biomechanical factors lead to meniscal damage, which subsequently exacerbates abnormal gait patterns, forming a self-reinforcing cycle that may accelerate joint degeneration and osteoarthritis progression. Several findings in the present study underscore this ambiguity. Notably, patients with Grade I–II injuries already demonstrated kinetic deficits comparable to those with Grade III injuries, raising the possibility that certain biomechanical alterations may precede structural progression rather than arise solely as a consequence of advanced damage. In addition, BMI differed significantly between groups and is independently associated with altered joint loading patterns; therefore, BMI may act not only as a covariate but also as a potential confounder influencing both gait mechanics and meniscal pathology. Similarly, although intergroup age differences were not statistically significant, the slightly higher mean age in the Grade III group compared to the control group warrants consideration. Age-related changes in neuromuscular control, muscle strength, and gait strategies could potentially influence biomechanical outcomes, even in the absence of statistical significance. This potential age-related confounding should be acknowledged as a limitation. Furthermore, the absence of data on lower limb alignment, detailed morphological characteristics, and pre-injury gait profiles limits our ability to clarify temporal and mechanistic pathways. Accordingly, the “stiff-knee” pattern described in this study should not be interpreted exclusively as a compensatory response to injury. It may alternatively represent a pre-existing or early maladaptive loading strategy that contributes to meniscal vulnerability. From a clinical perspective, this distinction is highly relevant: if abnormal biomechanics contribute to lesion development, gait retraining and load optimization may serve not only rehabilitative but also preventive roles. Longitudinal studies incorporating baseline biomechanical assessment prior to structural progression are required to resolve these causal relationships. The absence of data on joint reaction forces, electromyography, and biochemical markers further limits mechanistic insight. The study sample was also drawn from a single center, which may result in selection bias. Specifically, as participants were recruited from a tertiary hospital, consequently, the findings may not fully reflect the entire clinical spectrum of meniscal injuries. In addition, although disease duration was recorded for the injury groups, its relationship with gait parameters was not formally analyzed in the present study. As symptom duration may influence motor adaptation processes, patients with longer-standing symptoms could theoretically demonstrate either more pronounced biomechanical deficits due to progressive dysfunction or partial normalization resulting from compensatory adaptation. The absence of correlation analyses between symptom duration and gait variables limits our ability to interpret temporal adaptation patterns and should be considered when evaluating the findings. Although the sample size satisfied statistical requirements, it remains insufficient for subgroup analyses based on sex or exercise habits. Future prospective studies should analyze Stoller Grade I and II injuries separately using standardized radiological subclassification and structured data recording, recruit larger multicenter cohorts, and integrate multimodal assessments, including electromyography, wearable sensors, and inflammatory biomarkers, to more comprehensively capture neuromuscular and biological mechanisms and investigate potential gradient biomechanical differences between these subgrades; future studies should also systematically record analgesic and anti-inflammatory medication use to evaluate its potential impact on gait biomechanics.

## Conclusion

In conclusion, this study demonstrates that patients with meniscus injuries across different Stoller grades exhibit significant differences in gait kinematics and kinetics compared with healthy controls. Notably, even patients with Grade I–II injuries already show kinetic deficits similar to those observed in Grade III patients, whereas progressive increases in injury severity are associated with gradually exaggerated kinematic deviations. By elucidating the complex relationships between Stoller grading and gait characteristics, this study not only provides a functional complement to MRI diagnostics but also offers a biomechanical foundation for early functional assessment and the development of individualized rehabilitation strategies.

## Abbreviations

MRI: magnetic resonance imaging
GRF: ground reaction force
BMI: body mass index
OA: osteoarthritis
ACL: anterior cruciate ligament
MANCOVA: multivariate analysis of covariance
